# Clinical, diagnostic and epidemiological implications of *Hepatozoon* spp., *Babesia* spp. and *Leishmania infantum* infection in cats and dogs in a Mediterranean periurban setting

**DOI:** 10.1007/s00436-022-07705-2

**Published:** 2022-11-05

**Authors:** María Ortuño, Ana Bernal, Yaarit Nachum-Biala, Clara Muñoz, José Risueño, Juana Ortiz, Gad Baneth, Eduardo Berriatua

**Affiliations:** 1 Departamento de Sanidad Animal, Facultad de Veterinaria, Campus de Excelencia Internacional Regional ‘Campus Mare Nostrum’, Universidad de Murcia, Murcia, Spain; 2 Centro de Zoonosis del Ayuntamiento de Murcia, Carril Torre Molina, La Albatalia, Murcia, Spain; 3 Koret School of Veterinary Medicine, The Hebrew University of Jerusalem, Rehovot, Israel

**Keywords:** *Babesia*, Canine, Feline, *Hepatozoon*, *Leishmania*, Spain

## Abstract

*Hepatozoon* spp., *Babesia* spp. and *Leishmania infantum* are common parasites of dogs in Mediterranean countries and are less frequent in cats, particularly *Babesia* spp. and *L. infantum*. Moreover, there is limited information on coinfections between these parasites and on *L. infantum’s* distribution in blood, skin and lymphoid tissue in cats. We used PCR and DNA sequencing to investigate the prevalence of these parasites and the aetiology of *Hepatozoon* spp. and *Babesia* spp., in blood, skin, spleen and lymph node samples from up to 212 stray cats and 82 abandoned dogs in southeast Spain. All except 2 dogs were healthy; instead, 112 cats had clinical signs. The estimated PCR prevalences (95% confidence interval) were 25% (19–31%) *Hepatozoon felis* in cats, 13% (6–21%) *Hepatozoon canis* in dogs, 1% (0–4%) *Babesia vogeli* in dogs, 0% *Babesia* spp. in cats and 21% (15–26%) and 44% (33–55%) *L. infantum* in cats and dogs, respectively, and infections were not associated with each other. *Leishmania infantum* prevalence in lymphoid tissue was significantly higher in dogs than in cats (*p* < 0.001), and dogs had higher parasite loads than cats (*p* = 0.012). Moreover, *L. infantum* prevalence was significantly higher in the skin and lymphoid tissue compared to blood in infected, asymptomatic animals but it was similar in cats with clinical signs, which also had higher parasite loads compared to infected, asymptomatic cats (p < 0.05). The study highlights significant differences between sympatric dogs and cats with respect to the parasite infections investigated, as well as the need to examine both lymphoid tissue and skin samples to maximise the sensitivity of *L. infantum* infection diagnosis.

## Background

Dogs and cats harbour several vector-borne pathogens, and the incidence of these infections is considered to be increasing (Wright et al. 2020). Domestic animals are an integral part of society, and preserving their health is crucial to ensure their welfare and prevent the transmission of zoonotic pathogens to humans. The kinetoplastid protozoan *Leishmania infantum*, transmitted by phlebotomine sand flies, is a relevant veterinary problem with major public health implications in Mediterranean countries. The parasite targets mononuclear phagocyte system cells. Dogs are the domestic reservoir and the most susceptible species, and the disease may be associated with a wide range of clinical signs (Solano-Gallego et al. 2011). Other domestic and wild animal species may become infected and parasite reservoirs (Cardoso et al. 2021; Jiménez et al. 2014; Molina et al. 2012). Xenodiagnostic experiments have demonstrated the cat’s ability to transmit *L. infantum* to vectors (Maroli et al. 2007; Mendonça et al. 2020). Feline leishmaniosis (FeL) is being increasingly reported worldwide (Abramo et al. 2021), and it is similarly associated with a wide range of clinical signs, with a predominance for cutaneous lesions and lymphadenomegaly (Pennisi et al. 2015). *Leishmania infantum* was recently detected in a wild cat and other wildlife species in southern Spain, and an association was found between infections with *L. infantum* and *Hepatozoon* spp. and *Babesia* spp. in wild carnivores (Ortuño et al. 2021). The latter tick-borne apicomplexan protozoan parasites of vertebrates display a higher degree of host specificity and a comparatively low zoonotic potential (Springer et al. 2020). *Babesia* spp. infects erythrocytes and *Hepatozoon* spp. infects leukocytes. The severity of babesiosis and hepatozoonosis depends on the host species and may range from subclinical infections to severe illness (Baneth et al. 2011; Irwin 2010; Solano-Gallego & Baneth 2011).

The present study builds on the previously described work on leishmaniosis, hepatozoonosis and babesiosis in wildlife in southern Spain with the following aims: (i) estimating *L. infantum* prevalence in domestic stray cats by polymerase chain reaction (PCR) analysis of blood, skin and lymphoid tissue samples, (ii) assessing the aetiology and prevalence of *Hepatozoon* spp. and *Babesia* spp. in stray cats and abandoned dogs by PCR and DNA sequence analysis and (iii) investigating the statistical relationship between infections by the three parasites. We expected to provide clinical, diagnostic and epidemiological information on these infections in dogs and cats and on the extent to which *Hepatozoon* spp. and *Babesia* spp. species are shared between domestic animals and wildlife.

## Methods

### Study population and experimental design

The study was performed on blood, skin, spleen and/or lymph node DNA from samples from 294 animals, including 212 stray cats and 82 abandoned dogs from the city of Murcia metropolitan area in southeast Spain (37° 59′ 10″ N, 1° 07′ 49″ W). Abandoned dogs were animals whose owners refused to keep them and were either left in the street or taken to the municipal kennel. Those left in the streets are quickly removed by the local authorities and taken to the municipal kennel. In contrast, stray cats were animals born and raised in the street that had never been under human care.

The number of DNA samples analysed included 445 samples from cats (123 blood, 147 skin, 153 spleen and 22 lymph node DNA samples) and 287 samples from dogs (76 blood, 65 skin, 82 spleen and 64 lymph node DNA samples) (Table 1). The samples were part of a larger number collected between 2010 and 2016 for previous studies of randomly selected dogs and cats euthanised by the local authorities as part of a zoonosis control program. Samples here analysed included every one of those collected between 2010 and 2016 that had not been exhausted in previous studies. *Leishmania infantum* results from dogs were reported elsewhere (Ortuño et al. 2017; Risueño et al. 2012) and were used in the present study to investigate coinfections with *Hepatozoon* spp. and *Babesia* spp. and to compare dog and cat *L. infantum* infections.

**Table 1:** Percentage (number of positives/total analysed) of *Hepatozoon* spp., *Babesia* spp. and *Leishmania infantum* PCR-positive in cats and dogs

	*H. canis*	*H. felis*	*B. vogeli*	*L. infantum*					Skin + spleen + LN	Blood + skin + spleen + LN
	Blood	Blood	Blood	Skin	Blood	Lymphoid tissue				
						Spleen + LN	Spleen	LN		
Cats^a^	0 (0/123)	25 (31/123)	0 (0/123)	16 (24/147)	6 (7/123)	18 (31/175)	18 (28/153)	14 (3/22)	24 (43/176)	21 (44/212)
With clinical signs	0 (0/59)	29 (17/59)	0 (0/59)	10 (9/86)*	10 (6/59)**	14 (14/97)	14 (12/86)	18 (2/11)	16 (16/98)*	14 (16/112)*
Without clinical signs	0 (0/63)	22 (14/63)	0 (0/63)	23 (14/60)*	2 (1/63)**	22 (17/77)	24 (16/66)	9 (1/11)	34 (26/77)*	27 (27/99)*
Dogs^b^	13 (11/82)	0 (0/82)	1 (1/82)	12 (8/65)	4 (3/76)	42 (34/82)	33 (27/82)	22 (14/64)	44 (36/82)	44 (36/82)

*LN*, lymph node

^a^Clinical data from one cat was not available

^*^p < 0.05, ^s^ignificant differences; **p < 0.10, marginally significant differences

^b^Results of *L. infantum* infection in dogs (*N* = 82) from southeast Spain was published previously (Risueño et al., 2012; Ortuño et al., 2017) and included here for comparison with cat results

Animals were clinically examined pre-mortem, weighed and aged, and their body condition was estimated post-mortem. Age was assessed based on dentition (“How to determine a cat’s or dog’s age,” 1996), and cats were grouped accordingly into four categories: kittens (up to 1 year), young adults (2–6 years), mature adults (7–10 years) and senior (more than 10 years) (Quimby et al. 2021). For dogs, we considered a mean life expectancy of 11 years (Teng et al. 2022), and they were accordingly grouped as puppies (up to 9 months), young adults (from 9 months to 3 years), mature adults (from 4 to 8 years) and senior (more than 8 years) (Creevy et al. 2019). Similarly, three weight categories (small., medium and big) were considered: < 10 kg, 10–20 kg and > 20 kg for dogs and ≤ 3 kg, 3–4.5 kg and > 4.5 kg for cats. Animals were considered to be in low body condition if they had minimal or absent fat covering the ribs and the abdominal fat pad and had prominent ribs, lumbar vertebrae and pelvic bones (WSAVA 2020). Finally, dogs and cats were classified as pure or mongrel according to breed.

The studies had been approved by the University of Murcia’s ethical committee.

### DNA extraction and PCR amplification of *Hepatozoon *spp., *Babesia *spp. and *L. infantum *DNA

Dog and cat DNA were purified using a robot (Maxwell® Promega, Madison, WI, USA) and a commercial kit (Extractme Genomic DNA kit, Blirt©), respectively. Apicomplexan infection was firstly assessed by a generic end-point PCR amplifying a 360-base pair (bp) fragment of the 18S ribosomal RNA (rRNA) with forward Piroplasmid F (5′-CCA GCA GCC GCG GTA ATT C-3′) and reverse Piroplasmid R (5′-CTT TCG CAG TAG TTY GTC TTT AAC AAA TCT-3′) primers (Margalit Levi et al. 2018; Tabar et al. 2008). Positive samples were further analysed by a second PCR, this time with the specific primers forward PIRO A (5′-AAT ACC CAA TCC TGA CAC AGG G-3′) and reverse PIRO B (5′-TTA AAT ACG AAT GCC CCC AAC-3′) targeting a 408-bp 18S rRNA fragment of *Babesia* spp. (Barbosa et al. 2020; Olmeda et al. 1997) and *Hepatozoon* 18S forward (5′-GGT AAT TCT AGA GCT AAT ACA TGA GC-3′) and reverse (5′-ACA ATA AAG TAA AAA ACA YTT CAA AG-3′) primers targeting a 574-bp 18S rRNA fragment of *Hepatozoon* spp. (Almeida et al. 2012; Sarma et al. 2019). Positive control DNA was obtained from *Babesia* spp. or *Hepatozoon* spp. naturally infected dogs. DNA from a non-infected dog was used as a negative control, and ultra-pure water was employed as non-template DNA control.

*Leishmania infantum* DNA detection was carried out using a real-time PCR (rtPCR) assay with a Taqman probe, targeting kinetoplast minicircle DNA (kDNA). The protocols used were those of Mary et al. (2004) for dog samples and those of Dantas-Torres et al. (2017) for cat samples. The latter has a lower reaction volume and shorter running time (Dantas-Torres et al. 2017). Samples were tested in duplicate using 250 µg of template DNA per reaction and were deemed rtPCR-positive for cycle thresholds (CT) ≤ 38 in one or both reactions (Mackay 2007).

### *Hepatozoon* and *Babesia* DNA sequencing and phylogenetic analyses

*Hepatozoon* spp.- and *Babesia* spp.-positive PCR products were sequenced, and the BLAST tool (http://blast.ncbi.nlm.nih.gov/Blast.cgi) was used to compare with reference sequences in GenBank. They are now available in the GenBank database under the accession numbers MZ424831–MZ424837.

Phylogenetic trees were constructed using the MEGA X software (Kumar et al. 2018), and tree topology was inferred using the Neighbour-Joining method. Tree branches showing bootstrap values > 70% were considered confidential. Homologous sequences of *Sarcocystis cymruensis* (MG564723) and *Plasmodium falciparum* (accession number MF155937) were used as outgroups in *Hepatozoon* spp. and *Babesia* spp. phylograms, respectively.

### Statistical analysis

Prevalence was defined as the percentage of PCR-positives and was used to calculate 95% confidence intervals (95% CI). Fisher’s exact tests were used to compare the proportion of PCR-positives across independent demographic and clinical variables. Median CT of *L. infantum-*PCR-positive animals were similarly compared using the non-parametric Kruskal–Wallis test. For some comparisons, animals that were PCR-positive in more than one sample type were assigned the CT of the sample with the lowest value. The degree of agreement between PCR results in different biological samples from the same animals was analysed with the kappa statistic, considering > 0.81, 0.61–0.80, 0.41–60, 0.21–0.40, 0–0.20 and 0 as substantial, moderate, fair, slight and poor agreement, respectively (Thrusfield 2018). Associations were considered statistically significant for *p* < 0.05 and marginally significant for *p* < 0.10. The R software (http://cran.r-project.org/) was used for all analyses.

## Results

### Demographic data of cats and dogs

The cat population (percentage) included 105 males (50%) and 106 (50%) females and comprised 38 kittens (18%), 167 young adults (79%), 5 mature adults (2%) and 2 senior cats (1%). The dog population included 46 males (57%) and 35 females (43%) and was formed by 4 puppies (5%), 52 young adults (67%), 21 mature adults (27%) and 1 senior dog (1%). Concerning the size of cats and dogs, 99 (47%) and 23 (35%) were small, 95 (45%) and 21 (32%) were median and 17 (8%) and 22 (33%) were big, respectively. Finally, pure breeds and mongrels were present in 204 (96%) and 8 (4%) of cats and in 36 (44%) and 45 (56%) of dogs, respectively.

The following data were missing: sex data for one cat and one dog, age data for 4 dogs, size data for one cat and 16 dogs and breed data for one dog.

### Clinical signs in cats and dogs

Clinical signs were found in 112/211 (53%, 95% CI: 46–60%) cats and 2/81 (3%, 95% CI: 0–6%) dogs. The percentage (95% CI) of clinical signs in cats were low body condition in 33% (26–39%), lymphadenomegaly in 16% (11–21%), cutaneous lesions in 15% (10–20%), diarrhoea in 10% (6–13%), oral lesions in 7% (3–10%), ocular signs in 3% (1–5%), respiratory signs in 2% (0–4%), jaundice in 1% (0–1%) and pale mucous membranes in 1% (0–1%). Cutaneous clinical signs in cats included open skin lesions (11%, 7–16%), scabs (3%, 1–5%), exfoliative dermatitis (2%, 0–4%) and alopecia (0.5%, 0–1%). Whilst a formal post-mortem examination was not performed, splenomegaly and/or spleen lesions were detected in 5% (2–8%) of cats when samples from this organ were collected for *L. infantum* diagnosis. Clinical signs in dogs were alopecia, ulcerative dermatitis, lymphadenomegaly, low body condition and muscular atrophy in one dog, and pale mucous membranes and spleen lesions in the other dog.

### *Hepatozoon* spp. and *Babesia* spp. prevalence in blood samples

The percentage (95% CI) of *Hepatozoon* spp. PCR positives was 25% (19–31.0%) in cats and 13% (6–21%) in dogs. *Babesia* spp. was only detected in one dog (1%; 0–4%), a 4-year-old female Chow-Chow sampled in spring (Table 1).

The prevalence of *Hepatozoon* spp. infection in cats and dogs did not vary significantly according to gender, age, breed, size or the presence of clinical signs and was significantly higher in the summer in cats (*p* < 0.05) and in the winter in dogs (*p* < 0.05). No samples from dogs were collected in the summer.

### *Leishmania infantum* prevalence in blood, skin and lymphoid tissue samples

The overall PCR prevalence (95% CI) of *L. infantum* was 21% (15–26%) in cats and 44% (33–55%) in dogs (Table 1), and the median (range) CTs in PCR-positives were 35 (13–38) in cats and 32 (12–38) in dogs (*p* < 0.05). Prevalence overall, in the skin and in tissue (both lymphoid tissue and skin) samples, was higher in cats without clinical signs compared to those with clinical signs (*p* < 0.05). In blood samples, however, the prevalence was marginally higher in cats with clinical signs than in those without (*p* = 0.06). Moreover, overall prevalence was also marginally higher in big cats compared to other cats, and it was not associated with the animal’s gender, age or breed, and it was significantly higher in those sampled in winter compared to other times of the year (*p* < 0.01). Furthermore, *L. infantum* PCR prevalence and CTs in positive animals differed significantly depending on the sample type and between species for the same sample type.

In cats, PCR prevalence (95% CI) was 6% (2–10%) in blood, 14% (0–28%) in lymph nodes, 16% (10–22%) in the skin and 18% (12–24%) in the spleen (Table 1). In dogs, it was 4% (0–8%) in blood, 12% (4–20%) in the skin, 22% (12–32%) in lymph nodes and 33% (23–43%) in the spleen. Prevalence was higher in the skin and lymphoid tissue compared to blood in dogs and healthy cats, in lymphoid tissue in dogs compared to cats and in the skin in healthy cats compared to cats with clinical signs (*p* < 0.05) (Table 1). Moreover, CTs among lymphoid tissue-positive dogs were lower (indicating greater parasite load) than in lymphoid tissue-positive cats, in cats with clinical signs compared to those without and in mature adults compared to younger cats (*p* < 0.05).

All cats and dogs positive in blood were positive in skin, spleen and lymph node samples, except one dog that was positive in blood but not in the skin (Table 2). In contrast, results from skin, spleen and lymphoid tissue samples did not always coincide, particularly in cats, for which 7 animals were positive in the skin and negative in the spleen and vice versa (Table 2). In dogs, 2 and 4 animals were positive in the skin and negative in the spleen and lymph node, respectively, 9 and 12 animals were positive in the spleen and lymph node, respectively and negative in the skin and 5 were positive in the spleen and negative in lymph node and the opposite was the case for 7 animals (Table 2). Consequently, significant kappa coefficients reflected only fair agreement between PCR results in blood and skin and lymphoid tissue samples (*k* = 0.21–0.30), and either fair or moderate agreement between the skin and lymphoid tissues and between spleen and lymph nodes (*k* = 0.31–0.46) (Table 2).

**Table 2:** Number of animals with positive and negative *L. infantum* PCR results in different samples and kappa coefficient indicating the degree of agreement between test results

Sample (row/column)	Test result		
	Negative (neg)	Positive (pos)	Kappa coefficient
Cats			
Blood/skin			
Neg	54	19	
Pos	0	4	0.23
Blood/spleen			
Neg	56	17	
Pos	0	5	0.30
Blood/lymph node			
Neg	6	1	
Pos	0	1	0.60*
Skin/spleen			
Neg	110	12	
Pos	12	12	0.40
Dogs			
Blood/skin			
Neg	56	7	
Pos	1	1	0.21
Blood/spleen			
Neg	55	18	
Pos	0	3	0.25
Blood/lymph node			
Neg	50	12	
Pos	0	2	0.26
Skin/spleen			
Neg	48	9	
Pos	4	4	0.31
Skin/lymph node			
Neg	48	8	
Pos	2	6	0.46
Spleen/lymph node			
Neg	45	7	
Pos	5	7	0.42

^*^Not significant

### Relationship between *Hepatozoon* spp., *Babesia* spp. and *L. infantum* infections

There were no significant associations between parasitic infections in either dogs or cats. Mixed *Hepatozoon* spp. and *L. infantum* infections were detected in 12/205 (6%) of the animals, specifically in 6/82 (7%) of dogs and 6/123 (5%) of cats, and no animal was infected with all three parasites.

### Characterisation and phylogenetic analyses of *Hepatozoon* spp. and *Babesia* spp. DNA sequences

Three different *Hepatozoon* spp. sequences differing from one to three nucleotides were obtained from 11 PCR amplicons from dogs and were 100% identical to *H. canis* reference sequences (MN628317, MN628318, MN628320) and were named HC16, HC17 and HC18. Similarly, three *Hepatozoon* spp. sequences differing from one to three nucleotides from 31 PCR amplicons from cats were 99.7–100% identical to *H. felis* (MG386483, MG386484) and coded HF4, HF5 and HF6 (Table 3). The *Babesia* spp. sequence identified was 100% identical to *B. vogeli* (accession no. MK910150).

**Table 3:** Results of the BLAST analysis for the *Babesia* spp.- and *Hepatozoon* spp.-positive samples

New GenBank accession No	Isolate^a^	Parasite species	Domestic mammalian host (N)	Primers	Sequence length (bp)	Closest GenBank accession No	Identity (%)	Query cover (%)
MZ424831	BVo1	*Babesia vogeli*	Dog (1)	Piropl-FR	284	MK910150	100	100
MZ424832	HC16	*Hepatozoon canis*	Dog (5)	Piropl-FR	337	MN628317	100	100
MZ424833	HC17	*Hepatozoon canis*	Dog (2)	Piropl-FR	337	MN628318	100	100
MZ424834	HC18	*Hepatozoon canis*	Dog (4)	Piropl-FR	337	MN628320	100	100
MZ424835	HF4	*Hepatozoon felis*	Cat (29)	Piropl-FR	337	MG386483	100	100
MZ424836	HF5	*Hepatozoon felis*	Cat (1)	Piropl-FR	337	MG386484	100	100
MZ424837	HF6	*Hepatozoon felis*	Cat (1)	Piropl-FR	337	MG386484	99.7	100

^a^Isolate names are consecutive to those published in wildlife from Spain by Ortuño et al. (2021)

**Fig. 1:**
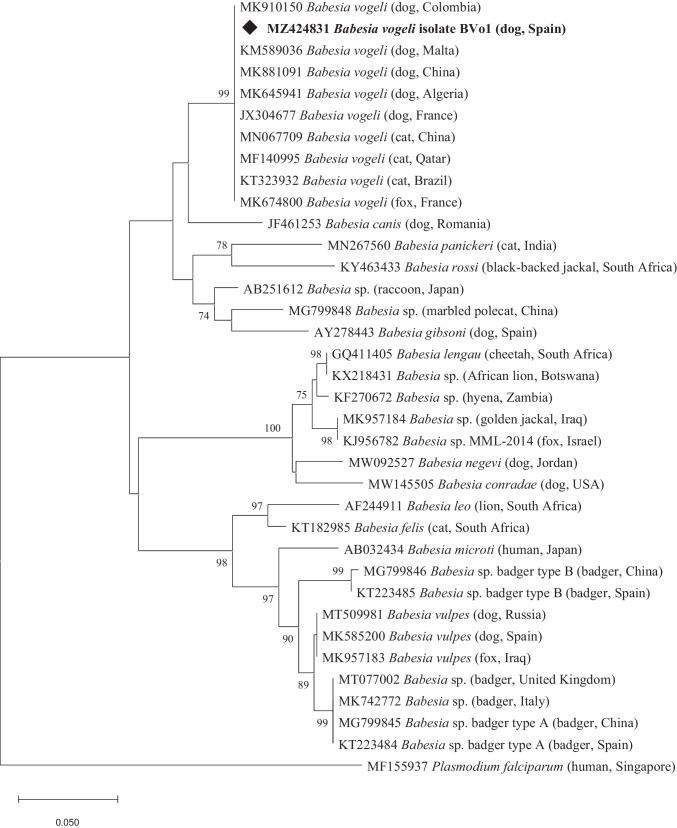
Phylogenetic tree of *Hepatozoon* spp. sequences from dogs and cats in this study (diamond-shaped) together with homologous sequences from GenBank. The evolutionary history was inferred by using the Neighbour-Joining method and the Kimura 2-parameter model. The percentage of trees in which the associated taxa clustered together is shown next to the branches. Bootstrap values below 70% are not shown. The tree is drawn to scale, with branch lengths measured in the number of substitutions per site. All positions with less than 95% site coverage were eliminated, i.e., fewer than 5% alignment gaps, missing data, and ambiguous bases were allowed at any position (partial deletion option). There were a total of 334 positions in the final dataset. Evolutionary analyses were conducted in MEGA X

*Hepatozoon canis* isolate HC16 was in the same cluster as isolates HC1 and HC9 from foxes in southern Spain, including Murcia Region, and also with *H. canis* found in foxes from France and in dogs from Algeria and India (71% bootstrap) (Fig. 1). Isolates HC17 and HC18 clustered with *H. canis* from dogs in Spain, Tunisia, Taiwan, India and Israel, cats and golden jackals from Israel, foxes from Murcia (HC2) and a beech marten from Andalucía (HC3), Spain (70% bootstrap) (Fig. 1).

*Hepatozoon felis* isolate HF4 clustered with *H. felis* from domestic cats in Spain, Israel, Italy and Angola (86% bootstrap) and a wild cat from Andalucía, Spain (HF1). Isolates HF5 and HF6 grouped with *H. felis* found in cats from Italy and Angola and in wild cats from Bosnia and Herzegovina, and also with *Hepatozoon ingwe* from an African leopard from South Africa, and separate from isolate HF2 found in wild cats from Murcia (Fig. 1).

**Fig. 2:**
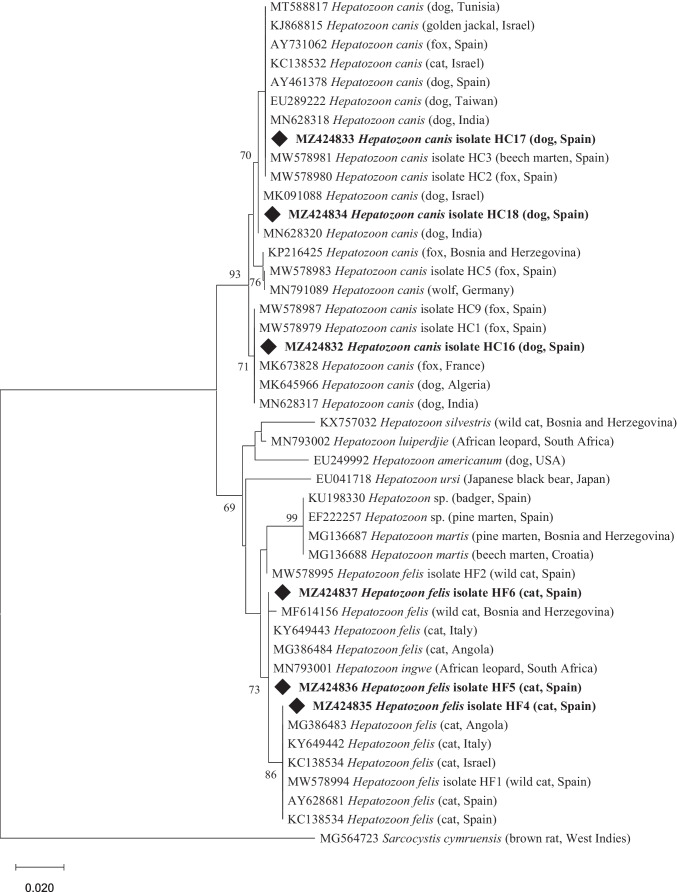
Phylogenetic tree of *Babesia* spp. sequences from dogs and cats in this study (diamond-shaped) together with homologous sequences from GenBank. The evolutionary history was inferred by using the Neighbour-Joining method and the Kimura 2-parameter model. The percentage of trees in which the associated taxa clustered together is shown next to the branches. Bootstrap values below 70% are not shown. The tree is drawn to scale, with branch lengths measured in the number of substitutions per site. All positions containing gaps and missing data were eliminated (complete deletion option). There were a total of 275 positions in the final dataset. Evolutionary analyses were conducted in MEGA X

In the phylogenetic analyses, *B. vogeli* from the dog (isolate BVo1) clustered with those in dogs from France, Malta, Colombia, Algeria and China, cats from Qatar, China and Brazil and foxes from France (99% bootstrap) (Fig. 2).

## Discussion

The *Hepatozoon* spp. DNA sequences in abandoned dogs and stray cats were in most cases identical to those in foxes, beech martens and wild cats in southern Spain (Ortuño et al. 2021), suggesting a common transmission cycle, as previously proposed for *L. infantum* (Ortuño et al. 2019). In contrast to wild carnivores in Spain (Ortuño et al. 2021) and cats in Cyprus (Attipa et al. 2017), *Hepatozoon* spp. infection was not associated with *L. infantum*. Potential synergism between leishmaniosis and other vector-borne infections in dogs and cats has been postulated (Baxarias et al. 2018; Cringoli et al. 2002; Mekuzas et al. 2009; Toepp et al. 2019), but the pathogenesis of apicomplexan parasites and *Leishmania* coinfections have not been established.

*Hepatozoon canis* and *B. vogeli* are transmitted by *Rhipicephalus sanguineus*, the most common tick in dogs in Spain (Checa et al. 2019; Estrada-Peña et al. 2017). The almost absence of *Babesia* spp. in dogs and cats suggests a low circulation of these parasites in the studied area and not because of low exposure to *R. sanguineus* since *H. canis* was found in 13% of dogs. Other articles have similarly reported low prevalences of *Babesia canis*, *B. vogeli* and *Babesia gibsoni* in dogs in Spain (Baxarias et al. 2018; Movilla et al. 2017; Tabar et al. 2009), whereas *Babesia vulpes* in dogs and foxes was more prevalent in northwest Spain and Portugal (Cardoso et al. 2013; Checa et al. 2019; García 2006; Miró et al. 2015). Feline babesiosis is rare worldwide and mostly restricted to South Africa (Jacobson et al. 2000). Vilhena et al. (2013) found 11% of *B. vogeli* PCR-positive cats in Portugal, and *Babesia* spp.-positive cats have been sporadically reported elsewhere in Europe and Asia (Penzhorn & Oosthuizen 2020).

The prevalence of *H. canis* in dogs and *H. felis* in cats reported here is higher than in most previous studies in the Iberian Peninsula, where it ranged from 0.7 to 3.3% for *H. canis* and 1.4 to 16% for *H. felis* (Díaz-Regañón et al. 2017; Maia et al. 2015; Movilla et al. 2017; Ortuño et al. 2008; Tabar et al. 2009; Vilhena et al. 2013). Albeit, Criado-Fornelio et al. (2006) and Dordio et al. (2021) reported 20% and 27% of *H. canis* and *H. felis* prevalence, respectively. The prevalence of these two apicomplexan species in other European countries was similarly variable (Cimpan et al. 2020; Licari et al. 2017; Otranto et al. 2017). In contrast to other studies from Spain and other countries in Europe (Criado-Fornelio et al. 2009; Díaz-Regañón et al. 2017; Giannelli et al. 2017), we did not detect *H. canis* or *Hepatozoon silvestris* in cats.

The risk of infection with *Hepatozoon* spp. depends on exposure to ticks and is conditioned by the animal’s lifestyle, use of ectoparasiticides and contact with wildlife (Pacifico et al. 2020). It is likely that most of the animals in this study, particularly stray cats, had never received ectoparasitic treatments and shared habitat with wildlife species and infected ticks. However, *H. felis* transmission routes are not known yet (Baneth et al. 2013), but ticks are suspected vectors. Congenital transmission may also occur, like for *H. canis* in dogs (Baneth et al. 2013; Murata et al. 1993). Predation was described for *Hepatozoon americanum* in dogs feeding on rodents and rabbits infected with a quiescent cystozoite form of the parasite (Johnson et al. 2009, 2008). Stray cats may engage more in predation than dogs and may ingest arthropod vectors when grooming. The time of the year when samples were collected could be an important factor in conditioning prevalence, and, in contrast to cats, no dogs were examined in the summer. Winter *Hepatozoon* spp. infections might be accounted for by transmission at this time of the year since *R. sanguineus* in Spain may also be active in the winter (Estrada-Peña et al. 2017) and/or infection becoming chronic for a long time (Baneth et al. 1998). In Israel, *Hepatozoon* spp. were most prevalent in cats during the winter (Baneth et al. 1998), but it was not seasonal in dogs (Baneth & Weigler 1997). In Iran, the prevalence of *Hepatozoon* spp. in dogs was highest in the summer (Barati & Razmi 2018).

*Leishmania infantum* was also strongly seasonal, and winter peaks in both dogs and cats would be associated with leishmaniosis’ long incubation period (Hernández et al. 2015; Oliva et al. 2006; Pennisi et al. 2012). Compared to other studies in the Iberian Peninsula, the prevalence of *L. infantum* in cats was slightly lower than the 26% to 30% found by Maia et al. (2008), Martín-Sánchez et al. (2007) and Millán et al. (2011) and higher than the 0% to 20% found in other studies (Alcover et al. 2021; Maia et al. 2010; Miró et al. 2014; Montoya et al. 2018; Sherry et al. 2011; Tabar et al. 2008). Prevalence estimations may differ depending on the number and lifestyle of cats sampled, the sample used for diagnosis, the time of the year and the animal’s clinical status and coinfections. Elevated parasitaemia is most typical in animals suffering clinical leishmaniosis, whilst in infected but healthy dogs, parasitaemia is less common than infection in the skin and lymphoid tissue (Chitimia et al. 2011), and according to our results, this would also apply to cats. Other studies comparing *L. infantum* prevalence in different cat samples have reported variable results. In stray and colony cats in Italy, PCR prevalence in blood was lower than in conjunctival swabs (Morganti et al. 2019) and marginally lower than in lymph node samples (Spada et al. 2020). In contrast, Chatzis et al. (2014) reported similar PCR prevalence in blood and tissue in healthy cats and a higher prevalence in tissues compared to blood in cats with skin lesions. It could be concluded that samples from skin and various lymphoid tissues, taken at different times of the year, are required for an accurate estimation of *L. infantum* infection prevalence in cat and dog populations.

The greater prevalence of infection in lymphoid tissue in dogs compared to cats and the similar prevalence in skin samples suggest that parasite visceralitation is lower in cats than in dogs and is compatible with cutaneous leishmaniosis being the most common clinical manifestation of the disease in cats (Solano-Gallego et al. 2007). However, in the present study, *L. infantum* infection in cats was not associated with clinical signs typical of FeL (Abramo et al. 2021; Pennisi et al. 2015). Still, none of the clinical signs detected is specific to FeL and may be due to numerous causes; for example, open skin wounds may be the result of fighting, which is common in stray cats. The differences between cats and dogs in infection prevalence and the parasite’s body distribution are remarkable. They are consistent with cats’ lower risk of leishmaniosis and having mostly cutaneous lesions, and they support the dogs’ role as a primary reservoir of infection. Notwithstanding this, the cats’ role in *L. infantum* transmission requires further investigation given that they are able to transmit the parasite to the vector, the prevalence may be relatively high, and cat populations are large. An objective assessment may only be possible in natural environments in the absence of dogs or other reservoirs of infection (Zanet et al. 2014).

Our *L. infantum* and *Hepatozoon* spp. studies support a common domestic and sylvatic transmission cycle for these parasites in southeast Spain (Ortuño et al. 2019, 2021), as well as the need to consider cats as a potentially important element in leishmaniosis epidemiology. It also highlights the importance of controlling stray cats and abandoned dog populations to reduce parasite prevalence, their impact on pets and the risk of zoonotic transmission.

## Conclusions

*Hepatozoon* spp. and *L. infantum* but not *Babesia* spp. are common parasites of stray cats and abandoned dogs in south-east Spain, and *Hepatozoon* spp. transmission cycles are likely to be shared with wildlife. A better demographic and sanitary control of this vulnerable population is required. Compared to dogs, cats have a lower probability of *L. infantum* infection and the parasite becoming latent in lymphoid tissue, and when it does, parasite concentration is lower. Differences between cats and dogs in *L. infantum* infection prevalence and body distribution have important epidemiological, diagnostic and clinical implications.

## Data Availability

The datasets supporting the conclusions of this article are included within the article. Raw data are available from the corresponding author upon reasonable request.

## Abbreviations

FeL: Feline leishmaniosis
PCR: Polymerase chain reaction
rRNA: Ribosomal RNA
rtPCR: Real-time PCR
kDNA: Kinetoplast minicircle DNA
CT: Cycle thresholds
